# Association between telomere length in peripheral blood leukocytes and risk of ischemic stroke in a Han Chinese population: a linear and non-linear Mendelian randomization analysis

**DOI:** 10.1186/s12967-020-02551-1

**Published:** 2020-10-12

**Authors:** Weijie Cao, Deqiang Zheng, Jie Zhang, Anxin Wang, Di Liu, Jinxia Zhang, Manjot Singh, Isinta Elijah Maranga, Mingyang Cao, Lijuan Wu, Manshu Song, Wei Wang, Youxin Wang

**Affiliations:** 1 Department of Epidemiology and Health Statistics, School of Public Health, Beijing Key Laboratory of Clinical Epidemiology, Capital Medical University, No. 10 Xitoutiao, Youanmen, Fengtai District, Beijing, 100069 China; 2 China National Clinical Research Center for Neurological Diseases, Beijing Tiantan Hospital, Capital Medical University, Beijing, 100050 China; 3 Department of Neurology, Beijing Tiantan Hospital, Capital Medical University, Beijing, 100050 China; 4 School of Medical and Health Sciences, Edith Cowan University, Perth, 6027 Australia

**Keywords:** Telomere length, Ischemic stroke, Mendelian randomization analysis, Disease biomarker

## Abstract

**Background:**

Many contradictory conclusions pertaining to the telomere length in peripheral leukocyte chromosomes as a potential biomarker for ischemic stroke (IS) risk have been reported by the various observational studies in previous years. This study aims to investigate whether the leukocyte telomere length is associated with an increased IS risk or not, based on the Mendelian randomization (MR) approach.

**Methods:**

Based on the NHGRI-EBI GWAS Catalog database, the Chinese online genetic database as well as the previous published studies, twelve single nucleotide polymorphisms (SNPs) with minor allele frequency ≥ 0.05 were selected and the leukocyte telomere length was measured in 431 first-ever IS patients and 304 healthy controls (quantitative polymerase chain reaction). To explore linear and non-linear effect of telomere length on the IS risk, we preformed the linear MR analysis (the inverse-variance weighted method, the maximum likelihood method, and the mode-based estimation method), and the non-linear MR analysis (semiparametric method with three tests for non-linearity, including the quadratic test, Cochran’s *Q* test, and the fractional polynomial test).

**Results:**

Two verified SNPs (rs11125529 and rs412658) were chosen as instrumental variables. In linear MR analysis, the adjusted odds ratios and 95% confidence intervals of IS for genetically predicted telomere lengths, based on the two SNPs, were 1.312 (0.979 to 1.759), 1.326 (0.932 to 1.888) and 1.226 (0.844 to 1.781) for the inverse-variance weighted method, the maximum likelihood method, and the mode-based estimation method, respectively. Three tests for nonlinearity failed to reject the null exactly, indicating that the relationship between telomere length and IS risk is unlikely to be non-linear.

**Conclusion:**

This MR study based on individual data does not provide strong evidence for a positive linear or non-linear effect of telomere length on the IS risk.

## Background

In China, stroke is one of the leading causes of death, and about 78% of all strokes are ischemic stroke (IS) [1]. IS is a complex syndrome triggered by cerebral embolism, with its major etiological subtypes being large artery stroke, cardioembolic stroke as well as the small vessel stroke [2]. Chronological age is one of critical factors in IS pathology but is not considered to be a causal factor [3]. This relationship might reflect the impact of biological aging, particularly, in regards to the accumulation of endothelial and vascular damage over time [4]. Telomeres are specialized DNA–protein complexes at linear chromosome ends in eukaryotes, and have been considered as a marker of biological aging at both the cellular and organism level [5, 6]. They safeguard genome stability by preventing DNA degradation and fusion at chromosomal termini [7]. The DNA component that include TTAGGG nucleotide repeats progressively shortens with each cell division. Telomere shortening to a critical length eventually induces cell senescence and programmed cell death, thereby they can be likened to a ‘mitotic clock’ reflecting the amount of cellular turnover within an individual [7]. Furthermore, the telomere attrition rate increased due to oxidative stress and inflammation, both of which are implicated as major mechanisms underlying biological aging [8–10]. Because of its presumed effect on biological aging, telomere length (TL) has been proposed as a potential biomarker for IS [11]. In addition to biological aging, TL shortening has been linked to various risk factors of stroke, including obesity, smoking, alcohol intake, psychological stress and depression [11].

Conventional observational studies have shown inconclusive associations of telomere shortening and the risk of stroke. Consistently, several original studies reported positive association between telomere shortening and the risk of stroke [12–14]. In contrast, evidence also suggests that shorter telomeres were not significantly associated with stroke risk [15–17]. However, meta-analysis have shown that there were less certain results to support the relationship between TL and stroke [18, 19].

Randomized controlled trials of interventions specific to TL have not yet been done in relation to stroke outcomes. In the absence of such trials, Mendelian randomization (MR) analysis focused on genetic instrument can be used to investigate the causality from routinely observational studies [20]. Based on two-sample MR design, three MR analyses have been performed to investigate the association between shorter telomeres and stroke risk and showed contradictory conclusions. First, a MR study showed that shorter telomeres lead to a marginally significantly decreased odds of stroke in individual-level data [21]. Another two MR studies suggested that TL may be a marker of IS and its subtypes rather than a cause [22, 23]. Two-sample MR typically assume that the exposure-outcome association is linear or log-linear [24]. Therefore, a non-linear TL-IS relationship, such as U-shaped, cannot be detected through this design. To tackle this nonlinearity problem, one-sample MR analysis can be used to explore the non-linear effect relating TL to IS [25]. In this present study, we performed one-sample MR analysis with individual-level data in a Han Chinese population to decipher the linear and non-linear causal role of TL in the IS risk, and to provide insight into the potential mechanisms.

## Methods

### Study population

All participants were recruited from Jidong Oil-field Hospital, Chinese National Petroleum, and Beijing Tiantan Hospital, Capital Medical University, during 2010–2013. A total of 755 participants aged 18 or above were found to be eligible. All participants who met any of the following criteria were excluded from the study: (1) history of mental illness or infectious disease; (2) history of aneurysm caused by cerebral infarction, cerebral haemorrhage or other cerebrovascular diseases, congenital heart disease, acute myocardial infarction, liver disease, renal failure, malignant tumour, chronic obstructive pulmonary disease, rheumatoid arthritis, or other diseases; and (3) pregnant or lactating women. All first‐ever IS patients were diagnosed according to the World Health Organization criteria [26]. In this study, 20 participants of non-Han Chinese descent were subsequently excluded. A total of 431 patients with IS and 304 healthy subjects were included in the final analysis.

This study was approved by the Ethics Committee of Capital Medical University, China (No. 2016SY23). This study was in accordance with the principles of the Declaration of Helsinki. All participants provided their written informed consent before taking part in this study.

### Leukocyte telomere length measurement

Blood samples were collected and processed according to the standardized protocol. Following 10 h. of overnight fasting, blood samples were collected by venipuncture in two different tubes containing an anticoagulant and a coagulant respectively. Samples were processed within 8 h. and stored at -80C until further measurement. Given that whole blood TL is a proxy for tissue-specific TL for many tissues, and blood TL is a proxy for TL in some tissues in epidemiological studies [27], in this study we measured TL in whole blood, but not different types of leukocytes.

Serum samples were used to measure the biochemical indices, and blood cells were used to extract the genomic DNA. Based on automated nucleic acid purification platform (BioTeke, AU1001-32, Beijing, China), genomic DNA was extracted from the 200 μl of frozen blood cells using the magnetic bead-based method for concentrating DNA (The Genomic DNA Magnetic Beads Kit, AU18016, BioTeke, Beijing, China). DNA concentration and purity were determined using a Nanodrop (ND-8000, Thermo Scientific, USA) and then normalized to 5 ng/μl in TE buffer (1 mM EDTA, 10 mM Tris–HCl [pH 8.0]). Absolute TL of each chromosome end of the peripheral leukocytes was measured by using a validated quantitative polymerase chain reaction (qPCR) on the method reported by O'Callaghan with modification [28, 29]. A single copy reference gene (*36B4*) which encodes the acidic ribosomal phosphoprotein P0, was used to normalize the quantity of the input DNA. Comparing telomere-to-*36B4* ratio to reference DNA of known TL reflects the absolute length of the telomeres. The master mix was prepared using Applied Biosystems reagents. Primer concentrations were: TelF 200 nM, TelR 200 nM, 36B4F 300 nM, and 36B4R 500 nM. The primer sequences (5′–3′) were as follows:

TelF CGGTTTGTTTGGGTTTGGGTTTGGGTTTGGGTTTGGGTT

TelR GGCTTGCCTTACCCTTACCCTTACCCTTACCCTTACCCT

36B4F CAGCAAGTGGGAAGGTGTAATCC

36B4R CCCATTCTATCATCAACGGGTACAA

Analysis of both telomere and 36B4 in experimental samples and reference DNA were run in triplicates using 5 ng DNA and both telomere and 36B4 amplifications were performed on the same run. A no template control of nuclease-free water was included in each run. In brief, qPCR was performed in 0.1 ml tubes on the Applied Biosystems QuantStudio 3 Real-Time PCR System (Thermo Scientific, USA), with the thermal cycling profile for both telomere and 36B4 amplifications: 2 min at 50 °C, 2 min at 95 °C, followed by 35 cycles of 95 °C for 15 s, 61 °C for 1 min, followed by a melt curve. The no template controls in all runs were no amplification. The melt curve should produce only one single distinct peak. Standard deviation for the Ct-value in replicates were less than 0.5. The inter-coefficients of variance for the Ct-value was less than 5%.

### Genotyping

Single nucleotide polymorphisms (SNPs) were selected from the NHGRI-EBI GWAS Catalog (https://www.ebi.ac.uk/gwas) and the online genetic database (https://www.ensembl.org) according to reported associations with TL and minor allele frequency (MAF) ≥ 0.05. Only one SNP (rs17653722) is reported in population of Chinese descent and other TL-related SNPs were based on the European ancestry. The exclusion of association could be confirmed in online software PhenoScanner (https://www.phenoscanner.medschl.cam.ac.uk/phenoscanner). Finally, twelve SNPs (rs11125529, rs6772228, rs10936599, rs7726159, rs9420907, rs17653722, rs3027234, rs8105767, rs409627, rs412658, rs6028466, rs755017) were selected for analysis. SNP genotyping was performed on these SNPs using the Sequenom Massarray iPLEX platform (Agena Bioscience, Inc., San Diego, CA, USA).

### Variables

Demographic characteristics of participants including age, gender, smoking, drinking, height, weight, body mass index (BMI), systolic blood pressure (SBP), diastolic blood pressure (DBP), were collected by questionnaire survey and physical examination. Biochemical data including fasting plasma glucose (FPG), triglycerides (TG), total cholesterol (TC), high‐density lipoprotein cholesterol (HDL‐C), low‐density lipoprotein cholesterol (LDL‐C), apolipoprotein A1 (ApoA1), apolipoprotein B (ApoB) were all tested via standard methods in the clinical laboratory of Beijing Tiantan Hospital. TL measurement and SNP genotyping were performed by laboratory personnel according to the standardized protocol.

### Statistical analysis

Demographic and clinical characteristics were represented as the mean ± standard deviation (SD) for continuous variables underlying the normal distribution; otherwise, the median (interquartile range) was used. Categorical variables were represented as frequency (percentage). The between-group differences of continuous variables were tested by the *t* test or the Mann–Whitney *U* test. The chi‐squared test was used to compare the proportions for categorical variables, and to test for Hardy–Weinberg equilibrium.

The genetic variant or genetic risk score (GRS) acts as an instrumental variable in MR analysis if: (1) they are truly associated with TL; (2) they are independent of other factors (confounders); and (3) they can only influence IS risk via the causal effect of the TL. All association of instrument variables with TL and other risk factors, which were susceptible to reverse association, were limited to health controls. Principal analyses assumed over-dominant effects (heterosis), with subsidiary analyses of other genetic models (dominant, recessive, co-dominant and additive model).

The *β* coefficients were obtained from the linear regression model with natural log-transformed TL (ln TL). The percentage difference in TL with risk genotype was obtained from [exp (*β*) − 1)] × 100. Then, the linear MR estimates for ln TL on the IS risk were calculated by the inverse-variance weighted method, the maximum likelihood method, and the mode-based estimation method, adjusting for age, sex, and other confounders (smoking status, drinking status, and levels of BMI, SBP, DBP, FPG, TG, TC, HDL-C, LDL-C, ApoA1, ApoB). All results were presented as the odds ratio (OR) of IS per 10% decrease in TL. Three tests for non-linearity of the semiparametric method (the fractional polynomial method and the piecewise linear method) were applied: the quadratic test, which assesses for a linear trend among the localised average causal effect (LACE) estimates, Cochran’s *Q* test, which assesses whether LACE estimates differ more than would be expected by chance, and the fractional polynomial test, which assesses whether the fractional polynomial model of degree 1 fits LACE estimates better than the linear model [25].

For all analyses, a two‐tailed *P* value < 0.05 was considered to be statistically significant. All statistical analyses were performed using R version 3.5.3 (R Foundation for Statistical Computing, Vienna, Austria).

## Results

### Participant characteristics

Demographic and clinical characteristics were described in Table 1. Among 735 participants (374 men and 361 women), the median age of the study population was 54 years (P_25_ 44 years, P_75_ 61 years) in all subjects, 47 years (35 to 59 years) in controls, and 57 years (49 to 63 years) in IS patients. Most of the IS patients were older males with smoking and drinking habits and higher weight, BMI, SBP, DBP, TG levels and lower TC, HDL‐C, ApoA1, ApoB levels. The levels of TL, height, FPG, LDL‐C, and ApoB/ApoA1 ratio were not statistically different between groups. There were no statistically significant differences in the genotype distribution of all SNP between the IS patients and the control group (Table 2). Four SNPs with MAF < 0.05 were excluded (MAF: 0.000 for rs6772228, 0.006 for rs9420907, 0.042 for rs3027234, and 0.002 for rs6028466). SNPs that deviated from Hardy–Weinberg equilibrium were excluded. Other eight SNPs, amongst the healthy controls, were all in accordance with the Hardy–Weinberg equilibrium.

**Table 1:** Characteristics of the study population

Parameters	Controls (*n* = 304)	Cases (*n* = 431)	*P*
Age, years	47 (35, 59)	57 (49, 63)	*1.736 × 10*^–19^*
Gender, *n *(%)			*2.404 × 10*^–4^*
Male	130 (42.80)	244 (56.60)	
Female	174 (57.20)	187 (43.40)	
Smoke, cigarettes/day, *n *(%)			*1.227 × 10*^–17^*
No	253 (83.20)	248 (57.50)	
1–10	29 (9.50)	41 (9.50)	
> 11	22 (7.20)	142 (32.90)	
Drink, *n *(%)			*8.893 × 10*^–5^*
No	221 (72.70)	252 (58.50)	
Yes	83 (27.30)	179 (41.50)	
TL, kb	6.74 (5.18, 8.43)	6.44 (4.88, 8.93)	0.144
Height, cm	165.0 (159.0, 172.0)	167.0 (160.0, 172.0)	0.324
Weight, kg	66.95 (57.10, 77.58)	70.00 (63.00, 77.80)	0.003*
BMI, kg/m^2^	24.31 (22.08, 27.16)	25.27 (23.07, 27.39)	0.003*
SBP, mmHg	124 (113, 136)	138 (125, 150)	*1.087 × 10*^–16^*
DBP, mmHg	79 (72, 88)	82 (76, 91)	*9.324 × 10*^–7^*
FPG, mmol/L	5.00 (4.70, 5.50)	5.17 (4.61, 6.00)	0.053
TG, mmol/L	1.26 (0.86, 1.80)	1.34 (1.00, 1.87)	0.017*
TC, mmol/L	4.50 (3.99, 5.05)	4.22 (3.47, 4.99)	*2.451 × 10*^–4^*
HDL‐C, mmol/L	1.19 (1.01, 1.38)	1.08 (0.91, 1.24)	*5.497 × 10*^–9^*
LDL‐C, mmol/L	2.53 (2.12, 2.92)	2.46 (1.92, 3.00)	0.232
ApoA1, g/L	1.26 (1.14, 1.36)	1.19 (1.07, 1.29)	*1.727 × 10*^–9^*
ApoB, g/L	0.98 (0.85, 1.19)	0.93 (0.73, 1.11)	*1.845 × 10*^–4^*
ApoB/ApoA1 ratio	0.79 (0.66, 1.01)	0.77 (0.61, 0.96)	0.198

*TL* telomere length, *BMI* body mass index, *SBP* systolic blood pressure, *DBP* diastolic blood pressure, *FPG* fasting plasma glucose, *TG* triglycerides, *TC* total cholesterol, *HDL‐C* high‐density lipoprotein cholesterol, *LDL‐C* low‐density lipoprotein cholesterol, *ApoA1* apolipoprotein A1, *ApoB* apolipoprotein B

* *P* < 0.05 is statistically significant

**Table 2:** Genotype distributions of SNPs in IS patients and controls

SNP identifier	Chr	Gene	Genotype	Controls	Cases	*P*	*P*_*HWE*_
rs11125529	2	*ACYP2*				0.433	1.000
			C/C	202 (66.40%)	305 (70.80%)		
			C/A	92 (30.30%)	112 (26.00%)		
			A/A	10 (3.30%)	14 (3.20%)		
rs10936599	3	*MYNN*				0.240	1.000
			T/T	103 (33.90%)	121 (28.10%)		
			T/C	148 (48.70%)	225 (52.20%)		
			C/C	53 (17.40%)	85 (19.70%)		
rs7726159	5	*TERT*				0.252	0.718
			C/C	114 (37.50%)	155 (36.00%)		
			C/A	147 (48.40%)	195 (45.20%)		
			A/A	43 (14.10%)	81 (18.80%)		
rs17653722	12	*KRT80*				0.800	0.654
			G/G	220 (72.40%)	302 (70.10%)		
			G/T	76 (25.00%)	117 (27.10%)		
			T/T	8 (2.60%)	12 (2.80%)		
rs8105767	19	*LOC112268248*				0.473	0.563
			A/A	159 (52.30%)	215 (49.90%)		
			A/G	125 (41.10%)	177 (41.10%)		
			G/G	20 (6.60%)	39 (9.00%)		
rs409627	19	*ZNF676*				0.639	0.304
			G/G	129 (42.40%)	189 (43.90%)		
			G/C	145 (47.70%)	192 (44.50%)		
			C/C	30 (9.90%)	50 (11.60%)		
rs412658	19	*ZNF676*				0.596	0.441
			C/C	130 (42.80%)	188 (43.60%)		
			C/T	143 (47.00%)	190 (44.10%)		
			T/T	31 (10.20%)	53 (12.30%)		
rs755017	20	*ZBTB46*				0.461	0.907
			A/A	102 (33.60%)	163 (37.80%)		
			A/G	147 (48.40%)	199 (46.20%)		
			G/G	55 (18.10%)	69 (16.00%)		

*IS* ischemic stroke, *SNP* single nucleotide polymorphism, *Chr* chromosome

*P* < 0.05 is statistically significant

*P*_*HWE*_ < 0.05 indicating that the SNP in control group was not satisfied Hardy–Weinberg equilibrium

### Association estimates for individual SNPs

**Fig. 1:**
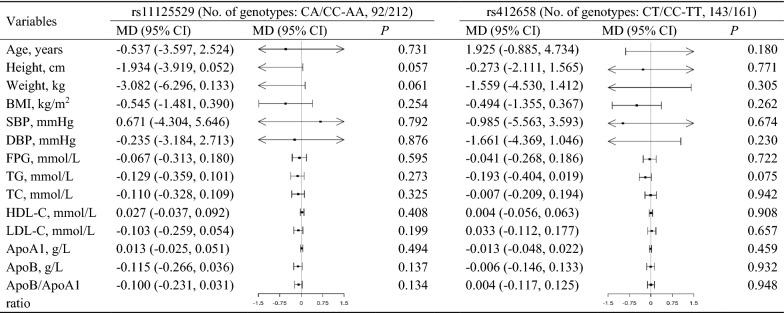
Associations of two single nucleotide polymorphisms related to telomere length with various characteristics in individuals free from known ischemic stroke at time of measurement. *MD* mean difference, *95% CI* 95% confidence interval, *BMI* body mass index, *SBP* systolic blood pressure, *DBP* diastolic blood pressure, *FPG* fasting plasma glucose, *TG* triglycerides, *TC* total cholesterol, *HDL‐C* high‐density lipoprotein cholesterol, *LDL‐C* low‐density lipoprotein cholesterol, *ApoA1* apolipoprotein A1, *ApoB* apolipoprotein B.

Association of each SNP with ln TL in all the assumed genetic models are shown in Additional file 1: Table S1 and Additional file 2: Table S2. To meet MR assumptions basically, two of the SNPs (rs11125529 and rs412658) were used as instrumental variables, and the unweighted GRS were constructed for non-linear MR analysis. By over-dominant model analysis, two SNPs in the control group were found to be associated with decreased TL, with risk genotype difference in ln TL of -0.108 (95% confidence interval (95% CI) − 0.204 to − 0.013) for rs11125529, − 0.089 (− 0.176 to − 0.001) for rs412658 (Table 3). These two TL-related SNPs were not associated with the conventional risk factors or other biochemical indicators in the control group (Fig. 1). Furthermore, these two SNPs displayed no direct evidence for an individual association with IS risk (*P* > 0.05). The ORs for IS of risk genotype (“short TL”) were 0.810 (0.580, 1.120) for rs11125529, and 0.890 (0.660 to 1.190) for rs412658 (Table 3).

**Table 3:** Candidate IVs and their association with TL and IS risk under over-dominant model

	Association with ln TL in controls		Association with IS in all subjects	
	MD (95% CI)	*P*	OR (95% CI)	*P*
rs11125529		0.026*		0.203
C/C-A/A	0.000 (0.000, 0.000)		0.000 (0.000, 0.000)	
C/A	− 0.108 (− 0.204, − 0.013)		0.810 (0.580, 1.120)	
rs412658		0.049*		0.428
C/C-T/T	0.000 (0.000, 0.000)		0.000 (0.000, 0.000)	
C/T	− 0.089 (− 0.176, − 0.001)		0.890 (0.660, 1.190)	

*TL* telomere length, *IS* ischemic stroke, *MD* mean difference, *95% CI* 95% confidence interval, *OR* Odds ratio

* *P* < 0.05 is statistically significant

### Association of TL with the IS risk

**Fig. 2:**
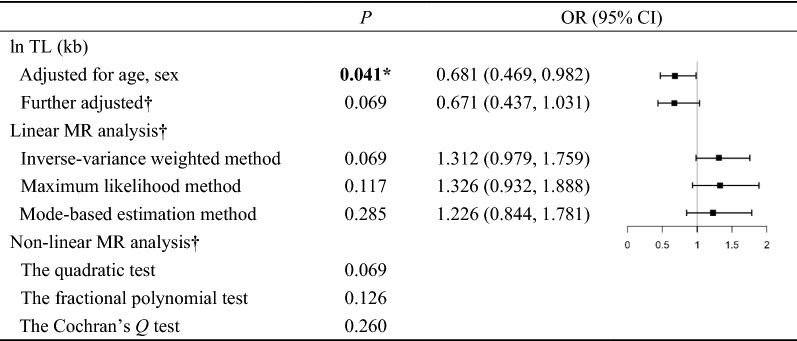
Estimates of association of telomere length (TL) and genetic prediction telomere with ischemic stroke risk. *P < 0.05 is statistically significant. † Corrected for regressions of TL and potential confounding factors, including age, sex, smoking status, drinking status, and levels of body mass index, systolic blood pressure, diastolic blood pressure, fasting plasma glucose, triglycerides, total cholesterol, high‐density lipoprotein cholesterol, low‐density lipoprotein cholesterol, apolipoprotein A1, apolipoprotein B. *OR* odds ratio, *95% CI* 95% confidence interval, *ln TL* Natural log-transformed telomere length, *MR* mendelian randomization.

In this case–control study, the OR (95% CI) for IS was 0.681 (0.469 to 0.982) adjusted for age and sex (Fig. 2). The association between TL and the IS risk was not statistically significant (OR (95% CI): 0.671 (0.437 to 1.031)) even after further adjusting for the smoking status, drinking status, and levels of BMI, SBP, DBP, FPG, TG, TC, HDL-C, LDL-C, ApoA1, ApoB.

**Fig. 3:**
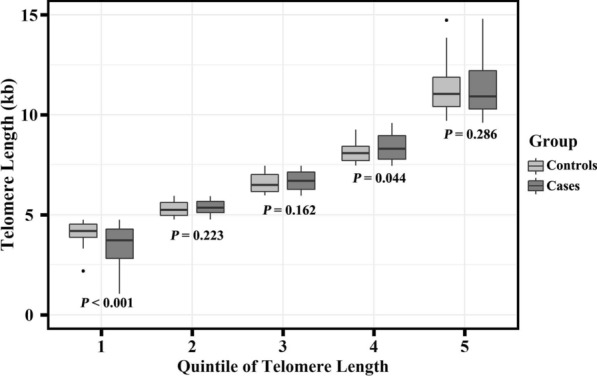
Box plot of telomere length across quintiles of the telomere length. *P* < 0.05 is statistically significant

Using rs11125529 and rs412658 as a proxy, the linear MR analysis provided no evidence of an overall association between genetically predicted TL and IS risk (OR (95% CI): 1.312 (0.979 to 1.759) for the inverse-variance weighted method, 1.326 (0.932 to 1.888) for the maximum likelihood method, and 1.226 (0.844 to 1.781) for the mode-based estimation method), after adjusting for the above-mentioned factors (Fig. 2). Using the unweighted GRS (rs11125529 and rs412658) as instrumental variables, three tests of nonlinearity MR analysis failed to reject the null hypothesis (*P* = 0.069 for the quadratic test, *P* = 0.126 for the fractional polynomial test, and *P* = 0.260 for the Cochran's *Q* test), indicating that the effect of telomere attrition on IS risk may not be non-linear. In Fig. 3, the box plot of TL between IS patients and controls across quintiles of the TL was also shown that there was no obvious non-linear TL-IS relationship. Two of five groups by the quintiles of TL, there were statistically significant differences in TL between the IS patients and the controls.

## Discussion

Based on linear and non-linear MR analysis, we used TL-related SNPs (or unweighted GRS) as proxies to clarify whether TL is causally relevant to IS or not. Our results showed no causal association between the genetically shortened TL and the increased IS risk.

Many epidemiologic studies have examined the relationship between TL and stroke risk, but results were not consistent enough. Shorter TL were not significantly associated with IS in the prospective and retrospective studies carried out in the Caucasian [15, 30, 31]. A nested case–control study including 504 case–control pairs of American female nurses showed a non-significant association between relative TL and IS (lowest vs. highest quartile: OR = 0.82, 95% CI 0.52–1.32, *P* = 0.42) [15]. Consistent with findings from the retrospective study, a prospective study (1,136 American adults aged 65 years and older at baseline, average 6.1 years of follow-up, 33 new cases of IS) reported that leukocyte TL change did not associate with IS (1st quartile vs. 4th quartile: relative risk (RR) = 1.61 (95% CI 0.46–5.68; *P* = 0.46) [30]. Similarly, in a cohort study including 4,576 Danish at baseline (10 years of follow-up, 295 new case of IS), the adjusted RR of IS for TL (4th quartile vs. 1st quartile) were 0.95 (95% CI 0.64–1.40; *P* = 0.60) [31]. In a case–control study carried out in Wuhan (capital of Hubei Province, South China), including 1,309 stroke patients and 1,309 age- and sex-matched control subjects, OR (95% CI) for IS risk were 2.12 (1.62–2.77) compared the first quartile (shortest) to the fourth (longest) quartile [14]. In another case–control study carried out in Shenzhen (Guangdong Province, South China) including 150 cases of IS and 150 siblings of patients free of stroke, shortened TL was independently associated with IS (OR = 4.00, 95% CI 1.28–12.77) [32]. The present MR did not provide strong evidence of causal association between short TL and IS, consistent with findings from the prospective studies and the retrospective studies carried out in the Caucasian, while different to case–control studies performed in the Chinese.

Despite previous retrospective observational studies reporting an TL-stroke association, such a relationship has not been firmly supported by evidence from prospective cohort studies. Subgroup analysis of four meta-analysis (stratified by study design) have shown consistent results that shorter TL were not significantly associated with stroke risk in prospective studies or in studies with a high quality score [18, 19, 33, 34]. In a recent meta-analysis, shorter TL was associated with a significant 11% higher risk for stroke (RR = 1.12, 95% CI 1.05–1.19), although with significant heterogeneity between studies (*I*^*2*^ = 81.1%, *P*_*het*_ < 0.001) [33]. After stratified by study design, the TL-IS relationship was not significance in those prospective studies (RR = 1.03, 95% CI 0.96–1.09) but remained significant in retrospective studies (RR = 1.81, 95% CI 1.54–2.13) [33]. MR analysis which simulates natural experiments based on genetic variants, is consider as an interface between cohort studies and the intervention trials at the evidence level [35]. The inconsistency might be explained by the confounding, reverse causation or recall bias, which might be avoided in MR analysis but not in observational studies.

Three linear MR studies based on two-sample MR design also presented inconsistent results in European ancestry. One MR analysis showed conflicting results which shorter TL was marginally statistically significantly associated with the decreased risk of stroke [21]. Another two linear MR analysis provided no evidence of the linear association between genetically predicted short TL and IS as well as its subtypes [22, 23]. Similarly, we also found null linear relationship between genetically predicted TL and IS risk in a Han Chinese population.

There are several possible explanations for the discrepancy between retrospective and prospective cohort studies. One hypothesis is that TL had causal effect on IS risk under certain circumstances. Difference in factors, such as participant age range, TL measurement technique, and so on, might influence the TL-IS relationship. A previous study indicate that the positive association between short TL and the risk of stroke or post-stroke death might only exist in the seniors population (ranging from 65 to 73 years old), therefore the effect of age might need to be taken into consideration in future studies [11]. However, people aged 65–73 years comprised a relatively low proportion of all participants (9.52%) in our research. Furthermore, although absolute TL measured by qPCR showed a strong correlation (*r*^*2*^ = 0.75, *P* < 0.0001) with the results obtained with terminal restriction fragment analysis (the gold standard for TL measurement). However, slightly difference in TL measurement could affect the TL-IS relationship [28].

Another hypothesis, based on epidemiological evidence, explaining this contradiction is that shorter TL is inversely associated with the risk of IS, which means that TL may be a downstream biological consequence of the IS onset. Figure 3 illustrates that no clear linear or non-linear relationship exists between TL and the risk of IS. However, not all of the differences between the TL and the IS risk were statistically significant within the different TL groups, indicating that there may be a feedback mechanism within certain TL range. Additionally, age is one of the major risk factors of TL and IS, so the effect of age needs to be excluded to prove this hypothesis [7, 11]. Estimates from previous MR studies and our study have avoided the possibility of inverse association to some extent, but more work is still needed to be determined the possible role of TL. For example, bidirectional MR analysis may be further used to orient the causal direction of TL-IS relationship [36]. Otherwise, in terms of the conflicting results from different research designs, other possibility is that there is no association between TL and IS risk. At present, however, the mechanism research on the relationship of TL and IS is still lacking and lagging, and we cannot rule out the possibility that the existence of certain compensation mechanisms may have affected our results.

For the chief strengths of our study, we explored the possible shape of the potential causal relation between TL and IS risk in a one-sample MR framework using linear and non-linear MR methodology. As a result of the MR analysis, potential reverse causality was eliminated and confounding bias was reduced because genetic instruments were not associated with individual risk factors that may affect results from conventional observational studies. Secondly, although the potential pleiotropic effects were unavoidable in this study, we searched comprehensively from genotype to phenotype to identify the potential pleiotropic effects and further provided possible evidence of the SNP instrument validity that the SNPs have no effect on available confounding factors, to reduce the likelihood of bias due to violation of the instrumental variable analysis. Furthermore, to our knowledge, this is the first linear and non-linear MR study assessing TL in relation to the IS risk in a Han Chinese population. Otherwise, healthy controls were randomly recruited from the general population covering same geographical area, which could decrease the selection bias of the results.

This study also has some limitations. Firstly, MR analysis has stringent assumptions [20]. Completely ruling out potentially pleiotropic effects or an additional biological causal pathway is a challenge for all MR analyses. We are limited by current knowledge and other unavailable confounders, so we cannot exclude the possibility that our estimates are biased by currently unknown pleiotropic effects. Secondly, insufficient statistical power was a common limitation of one-sample MR analysis, and therefore we cannot exclude type II error as an explanation for the null results [37]. Our study does not provide strong evidence for a positive linear or non-linear effect of TL on IS, but does not rule out that genetically predicted TL by unidentified genetic instruments might play a role. Finally, our study was conducted in middle to early late aged participants of Han Chinese descent based on Northern China. Further MR research needs to be explored in a larger and more representative samples, including those from a non-Asian ethnicity.

## Conclusion

In conclusion, although TL is associated with the risk of IS based on the conventional case–control analysis, this one-sample MR study suggests that negative association of TL with the IS risk is unlikely to be linear or non-linear causal. There is a need to identify the specific genetic, biochemical, and environmental biological mechanisms responsible for this association. Further research with larger sample sizes, which will be able to perform stratified analysis by age or other strong risk factors, is necessary to understand the causal pathways underpinning this association.

## Supplementary information

**Additional file 1: Table S1.** Association between SNP genotypes and telomere length under co-dominant and additive model.

**Additional file 1: Table S2.** Association between SNP genotypes and telomere length under dominant, recessive and over-dominant model.

## Data Availability

The datasets used and/or analyzed during the current study are available from the corresponding author on reasonable request.

## Abbreviations

ApoA1: Apolipoprotein A1
ApoB: Apolipoprotein B
BMI: Body mass index
DBP: Diastolic blood pressure
FPG: Fasting plasma glucose
GRS: Genetic risk score
HDL-C: High‐density lipoprotein cholesterol
IS: Ischemic stroke
LACE: Localised average causal effect
LDL-C: Low‐density lipoprotein cholesterol
ln TL: Natural log-transformed telomere length
MAF: Minor allele frequency
MR: Mendelian randomization
OR: Odds ratio
qPCR: Quantitative polymerase chain reaction
RR: Relative risk
SBP: Systolic blood pressure
SD: Standard deviation
SNPs: Single nucleotide polymorphisms
TC: Total cholesterol
TG: Triglycerides
TL: Telomere length
95% CI: 95% confidence interval
