# Supplementary material for: Association between telomere length in peripheral blood leukocytes and risk of ischemic stroke in a Han Chinese population: a linear and non-linear Mendelian randomization analysis
Source: J Transl Med. 2020 Oct 12;18:385. doi: 10.1186/s12967-020-02551-1 (PMC7552559; doi:10.1186/s12967-020-02551-1)
Supplement: Supplementary file 1 — Additional file 1: Table S1. Association between SNP genotypes and telomere length under co-dominant and additive model. [file 12967_2020_2551_MOESM1_ESM.docx]

**Table S1 Association between SNP genotypes and telomere length under** **co-dominant and additive model**

| SNP identifier | Genotype | *n* | Co-dominant model | |  | Additive model | |
| --- | --- | --- | --- | --- | --- | --- | --- |
|  |  |  | MD (95% CI) | *P* |  | MD (95% CI) | *P* |
| rs11125529 |  |  |  | 0.084 |  | -0.068 (-0.148, 0.013) | 0.099 |
|  | C/C | 202 | 0.000 (0.000, 0.000) |  |  |  |  |
|  | C/A | 92 | -0.108 (-0.204, -0.012) |  |  |  |  |
|  | A/A | 10 | 0.008 (-0.239, 0.255) |  |  |  |  |
| rs10936599 |  |  |  | 0.799 |  | -0.021 (-0.084, 0.042) | 0.509 |
|  | T/T | 103 | 0.000 (0.000, 0.000) |  |  |  |  |
|  | T/C | 148 | -0.016 (-0.115, 0.082) |  |  |  |  |
|  | C/C | 53 | -0.044 (-0.174, 0.085) |  |  |  |  |
| rs7726159 |  |  |  | 0.359 |  | 0.044 (-0.020, 0.109) | 0.181 |
|  | C/C | 114 | 0.000 (0.000, 0.000) |  |  |  |  |
|  | C/A | 147 | 0.063 (-0.033, 0.158) |  |  |  |  |
|  | A/A | 43 | 0.077 (-0.061, 0.214) |  |  |  |  |
| rs17653722 |  |  |  | 0.846 |  | 0.006 (-0.080, 0.092) | 0.888 |
|  | G/G | 220 | 0.000 (0.000, 0.000) |  |  |  |  |
|  | G/T | 76 | 0.022 (-0.080, 0.124) |  |  |  |  |
|  | T/T | 8 | -0.050 (-0.326, 0.227) |  |  |  |  |
| rs8105767 |  |  |  | 0.590 |  | -0.030 (-0.102, 0.041) | 0.405 |
|  | A/A | 159 | 0.000 (0.000, 0.000) |  |  |  |  |
|  | A/G | 125 | -0.048 (-0.140, 0.044) |  |  |  |  |
|  | G/G | 20 | -0.026 (-0.208, 0.156) |  |  |  |  |
| rs409627 |  |  |  | 0.392 |  | -0.025 (-0.093, 0.043) | 0.473 |
|  | G/G | 129 | 0.000 (0.000, 0.000) |  |  |  |  |
|  | G/C | 145 | -0.063 (-0.155, 0.030) |  |  |  |  |
|  | C/C | 30 | -0.005 (-0.161, 0.150) |  |  |  |  |
| rs412658 |  |  |  | 0.123 |  | -0.015 (-0.083, 0.052) | 0.661 |
|  | C/C | 130 | 0.000 (0.000, 0.000) |  |  |  |  |
|  | C/T | 143 | -0.080 (-0.173, 0.012) |  |  |  |  |
|  | T/T | 31 | 0.043 (-0.109, 0.196) |  |  |  |  |
| rs755017 |  |  |  | 0.840 |  | 0.018 (-0.045, 0.080) | 0.583 |
|  | A/A | 102 | 0.000 (0.000, 0.000) |  |  |  |  |
|  | A/G | 147 | 0.026 (-0.073, 0.125) |  |  |  |  |
|  | G/G | 55 | 0.032 (-0.096, 0.161) |  |  |  |  |

All assumed genetic models were based on the control group. *P* < 0.05 is statistically significant.

SNP, Single nucleotide polymorphism; MD, Mean difference; 95% CI, 95% Confidence interval.
