# Supplementary material for: Association between telomere length in peripheral blood leukocytes and risk of ischemic stroke in a Han Chinese population: a linear and non-linear Mendelian randomization analysis
Source: J Transl Med. 2020 Oct 12;18:385. doi: 10.1186/s12967-020-02551-1 (PMC7552559; doi:10.1186/s12967-020-02551-1)
Supplement: Supplementary file 2 — Additional file 1: Table S2. Association between SNP genotypes and telomere length under dominant, recessive and over-dominant model. [file 12967_2020_2551_MOESM2_ESM.docx]

**Table S2 Association between SNP genotypes and telomere length under** **dominant, recessive and over-dominant model**

| SNP identifier | Dominant model | | | |  | Recessive model | | | |  | Over-dominant model | | | |
| --- | --- | --- | --- | --- | --- | --- | --- | --- | --- | --- | --- | --- | --- | --- |
|  | Genotype | *n* | MD (95% CI) | *P* |  | Genotype | *n* | MD (95% CI) | *P* |  | Genotype | *n* | MD (95% CI) | *P* |
| rs11125529 |  |  |  | **0.042*** |  |  |  |  | 0.739 |  |  |  |  | **0.026*** |
|  | C/C | 202 | 0.000 (0.000, 0.000) |  |  | C/C-C/A | 294 | 0.000 (0.000, 0.000) |  |  | C/C-A/A | 212 | 0.000 (0.000, 0.000) |  |
|  | C/A-A/A | 102 | -0.097 (-0.189, -0.004) |  |  | A/A | 10 | 0.042 (-0.205, 0.289) |  |  | C/A | 92 | -0.108 (-0.204, -0.013) |  |
| rs10936599 |  |  |  | 0.615 |  |  |  |  | 0.558 |  |  |  |  | 0.975 |
|  | T/T | 103 | 0.000 (0.000, 0.000) |  |  | T/T-T/C | 251 | 0.000 (0.000, 0.000) |  |  | T/T-C/C | 156 | 0.000 (0.000, 0.000) |  |
|  | T/C-C/C | 201 | -0.024 (-0.117, 0.069) |  |  | C/C | 53 | -0.035 (-0.151, 0.081) |  |  | T/C | 148 | -0.001 (-0.089, 0.087) |  |
| rs7726159 |  |  |  | 0.156 |  |  |  |  | 0.522 |  |  |  |  | 0.355 |
|  | C/C | 114 | 0.000 (0.000, 0.000) |  |  | C/C-C/A | 261 | 0.000 (0.000, 0.000) |  |  | C/C-A/A | 157 | 0.000 (0.000, 0.000) |  |
|  | C/A-A/A | 190 | 0.066 (-0.025, 0.156) |  |  | A/A | 43 | 0.041 (-0.085, 0.168) |  |  | C/A | 147 | 0.042 (-0.046, 0.130) |  |
| rs17653722 |  |  |  | 0.762 |  |  |  |  | 0.694 |  |  |  |  | 0.647 |
|  | G/G | 220 | 0.000 (0.000, 0.000) |  |  | G/G-G/T | 296 | 0.000 (0.000, 0.000) |  |  | G/G-T/T | 228 | 0.000 (0.000, 0.000) |  |
|  | G/T-T/T | 84 | 0.015 (-0.083, 0.114) |  |  | T/T | 8 | -0.055 (-0.330, 0.220) |  |  | G/T | 76 | 0.024 (-0.078, 0.125) |  |
| rs8105767 |  |  |  | 0.317 |  |  |  |  | 0.956 |  |  |  |  | 0.323 |
|  | A/A | 159 | 0.000 (0.000, 0.000) |  |  | A/A-A/G | 284 | 0.000 (0.000, 0.000) |  |  | A/A-G/G | 179 | 0.000 (0.000, 0.000) |  |
|  | A/G-G/G | 145 | -0.045 (-0.133, 0.043) |  |  | G/G | 20 | -0.005 (-0.183, 0.173) |  |  | A/G | 125 | -0.045 (-0.135, 0.044) |  |
| rs409627 |  |  |  | 0.246 |  |  |  |  | 0.714 |  |  |  |  | 0.171 |
|  | G/G | 129 | 0.000 (0.000, 0.000) |  |  | G/G-G/C | 274 | 0.000 (0.000, 0.000) |  |  | G/G-C/C | 159 | 0.000 (0.000, 0.000) |  |
|  | G/C-C/C | 175 | -0.053 (-0.142, 0.036) |  |  | C/C | 30 | 0.028 (-0.120, 0.175) |  |  | G/C | 145 | -0.061 (-0.149, 0.026) |  |
| rs412658 |  |  |  | 0.200 |  |  |  |  | 0.250 |  |  |  |  | **0.049*** |
|  | C/C | 130 | 0.000 (0.000, 0.000) |  |  | C/C-C/T | 273 | 0.000 (0.000, 0.000) |  |  | C/C-T/T | 161 | 0.000 (0.000, 0.000) |  |
|  | C/T-T/T | 174 | -0.058 (-0.147, 0.031) |  |  | T/T | 31 | 0.085 (-0.060, 0.231) |  |  | C/T | 143 | -0.089 (-0.176, -0.001) |  |
| rs755017 |  |  |  | 0.561 |  |  |  |  | 0.774 |  |  |  |  | 0.742 |
|  | A/A | 102 | 0.000 (0.000, 0.000) |  |  | A/A-A/G | 249 | 0.000 (0.000, 0.000) |  |  | A/A-G/G | 157 | 0.000 (0.000, 0.000) |  |
|  | A/G-G/G | 202 | 0.028 (-0.065, 0.121) |  |  | G/G | 55 | 0.017 (-0.098, 0.131) |  |  | A/G | 147 | 0.015 (-0.073, 0.103) |  |

All assumed genetic models were based on the control group. * *P* < 0.05 is statistically significant. SNP, Single nucleotide polymorphism; MD, Mean difference; 95% CI, 95% Confidence interval.
